# Sensing surface PEGylation with microcantilevers

**DOI:** 10.3762/bjnano.1.2

**Published:** 2010-11-22

**Authors:** Natalija Backmann, Natascha Kappeler, Thomas Braun, François Huber, Hans-Peter Lang, Christoph Gerber, Roderick Y H Lim

**Affiliations:** 1 National Centre of Competence in Research in Nanoscale Science, Department of Physics, University of Basel, Klingelbergstrasse 82, 4056 Basel, Switzerland; 2 Center for Cellular Imaging and Nanoanalytics, Biozentrum, University of Basel, Mattenstrasse 26, 4058 Basel, Switzerland; 3 Biozentrum and the Swiss Nanoscience Institute, University of Basel, Klingelbergstrasse 70, 4056 Basel, Switzerland

**Keywords:** AFM, cantilever sensor, polyethylene glycol, polymer brush, reversible collapse, static mode

## Abstract

Polymers are often used to modify surface properties to control interfacial processes. Their sensitivity to solvent conditions and ability to undergo conformational transitions makes polymers attractive in tailoring surface properties with specific functionalities leading to applications in diverse areas ranging from tribology to colloidal stability and medicine. A key example is polyethylene glycol (PEG), which is widely used as a protein-resistant coating given its low toxicity and biocompatibility. We report here a microcantilever-based sensor for the *in situ* characterization of PEG monolayer formation on Au using the “grafting to” approach. Moreover, we demonstrate how microcantilevers can be used to monitor conformational changes in the grafted PEG layer in different solvent conditions. This is supported by atomic force microscope (AFM) images and force–distance curve measurements of the microcantilever chip surface, which show that the grafted PEG undergoes a reversible collapse when switching between good and poor solvent conditions, respectively.

## Introduction

Polyethylene glycol (PEG) is often used as a protein-resistant surface layer in biomedicine and biotechnology on account of its good solubility in water and low toxicity. Polypeptide drugs and nanoparticles designed for drug delivery exhibit enhanced biocompatibility and proteolytic resistance (e.g., “stealth” particles) when modified with PEG (i.e., PEGylation) [1–2]. PEG-functionalized surfaces are highly effective at reducing protein adsorption from blood [3] thereby improving the biocompatibility of biomedical implants [4]. The protein-resistant properties of PEG have also been applied to reduce membrane fouling in ultrafiltration membranes for water purification [5].

It is important to develop a technique that can provide a fast, real-time characterization of layer formation as well as detect conformational changes in the obtained polymer layer. For instance, an understanding of how different environmental conditions such as solvent quality or protein concentration in solution can affect the conformation of a surface-grafted PEG layer, and how this might influence its interfacial properties may prove beneficial towards optimizing the PEGylation process. Although techniques such as X-ray photoelectron spectroscopy (XPS) [6–7] and ellipsometry [8] have been proven powerful in terms of studying layer formation, a direct determination of local morphology/conformation changes in the polymer layer is largely restricted to nanomechanics-based techniques such as the surface force apparatus [9] and atomic force microscopy (AFM) [10]. Developments in the quartz crystal microbalance with dissipation (QCM-D) [11] and surface plasmon resonance (SPR) [12] also allow for the characterization of adsorption-induced and structural changes in interfacial polymer layers.

The high sensitivity of microcantilever sensors has proven to be a powerful platform for detecting molecular interactions in a label-free, time resolved manner [13–14]. By an asymmetrical chemisorption of molecules (i.e., on one side of the microcantilever), the sensors can detect processes in “static” mode by measuring the bending of a microcantilever due to stress formation during the adsorption process; or in “dynamic” mode where the resonant frequency of an oscillating microcantilever shifts due to mass adsorption on its surface. The versatility of the microcantilever technique as a chemical/biological sensor has been demonstrated for vapors [15], ions [16], DNA [17–18], proteins [19–20], antibiotics [21] and pathogenic microorganisms [22–23]. The mechanical sensitivity of the static mode technique stems from changes in surface stress caused by molecular interactions with the surface (change in the electronic charge distribution of the substrate’s surface atoms) [24] and by lateral interactions within the molecular layer (electrostatic forces, structural changes and steric competition) [14]. This sensitivity to structural changes in static mode operation has shown to be particularly suited for measuring binding processes based on conformational changes of molecules attached to the microcantilever’s surface such as proteins [25–26], DNA [27] or lipid bilayers [28]. Recently, Bumbu et al. [29] applied the static mode technique to study the behavior of poly(methyl methacrylate) brushes that had been polymerized from the silicon surface of a microcantilever sensor, i.e., using a “grafting from” approach. While this allowed the authors to study the *in situ* swelling and collapse of poly(methyl methacrylate) brushes, the kinetics of brush formation could not be monitored in real-time.

The driving impetus behind this work is to apply microcantilever sensors operated in static mode to study in real-time (1) the kinetic aspects of surface PEGylation, and (2) conformational changes in the PEG layer over a timescale of tens of minutes *in situ*. Specifically, thiol-terminated PEG (mPEG–SH, 20 kDa) chains have been covalently tethered onto Au-coated microcantilever surfaces by the “grafting to” approach. When switching between good (phosphate-buffered saline buffer, PBS) and poor solvent conditions (a binary mixture of 20% 2-propanol in PBS), we observe a marked response that is characteristic of a reversible collapse in the PEG layer.

## Results

**Figure 1:**
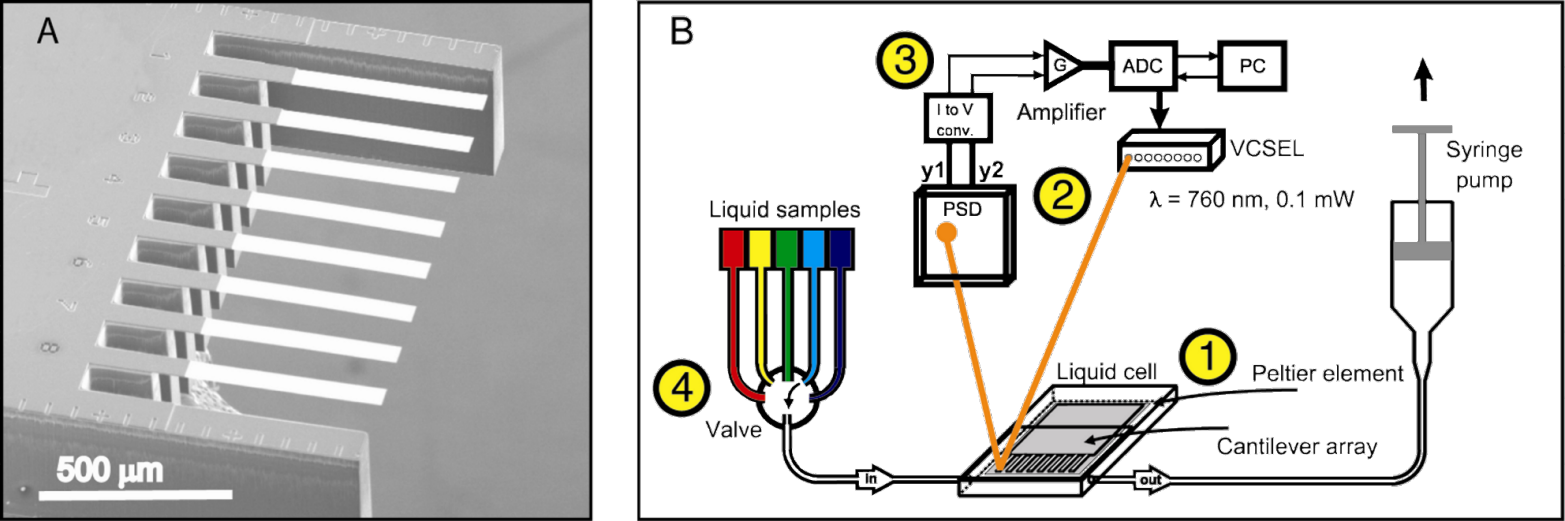
(A) Scanning electron microscope image of a silicon microcantilever array consisting of eight cantilevers and two sidebars. (B) Schematic drawing of the sensor instrument: 1 - the measurement cell with a mounted microcantilever array, 2 – optical-read out system comprising vertical cavity surface emitting lasers (VCSELs) and a position sensitive detector (PSD), 3 – data processing and acquisition, 4 – valve selector connected to liquid samples.

**PEGylation of Au-coated microcantilevers.** In our study we applied a direct “grafting to” approach where mPEG–SH was covalently bound onto the Au surface from solution. Each microcantilever in the eight-cantilever array (Figure 1A) was asymmetrically coated with Au to favor only the covalent binding of thiolated (mPEG–SH) molecules to that microcantilever surface. Prior to our experiments, two of eight microcantilevers in a Au-coated array were passivated (i.e., blocked) with monolayers of undecanethiol exposing four ethylene glycol units (EG_4_–C_11_–SH). These were used as internal reference microcantilevers within the same array (see Experimental section). This step is required to exclude any signal drift caused by external influences such as buffer mixing effects, non-specific binding on the lower side of the microcantilever as well as temperature and refractive index changes [30].

To study surface PEGylation on the remaining six microcantilevers, the array was mounted into the measurement cell and placed in an optical read-out system that detects the bending in each microcantilever via the deflection of an external laser beam focused at the apex of each microcantilever (Figure 1B). By definition, a downward deflection of the microcantilever (opposite to the Au-coated surface) is caused by compressive stress and an upward bending of the microcantilever describes tensile stress.

**Figure 2:**
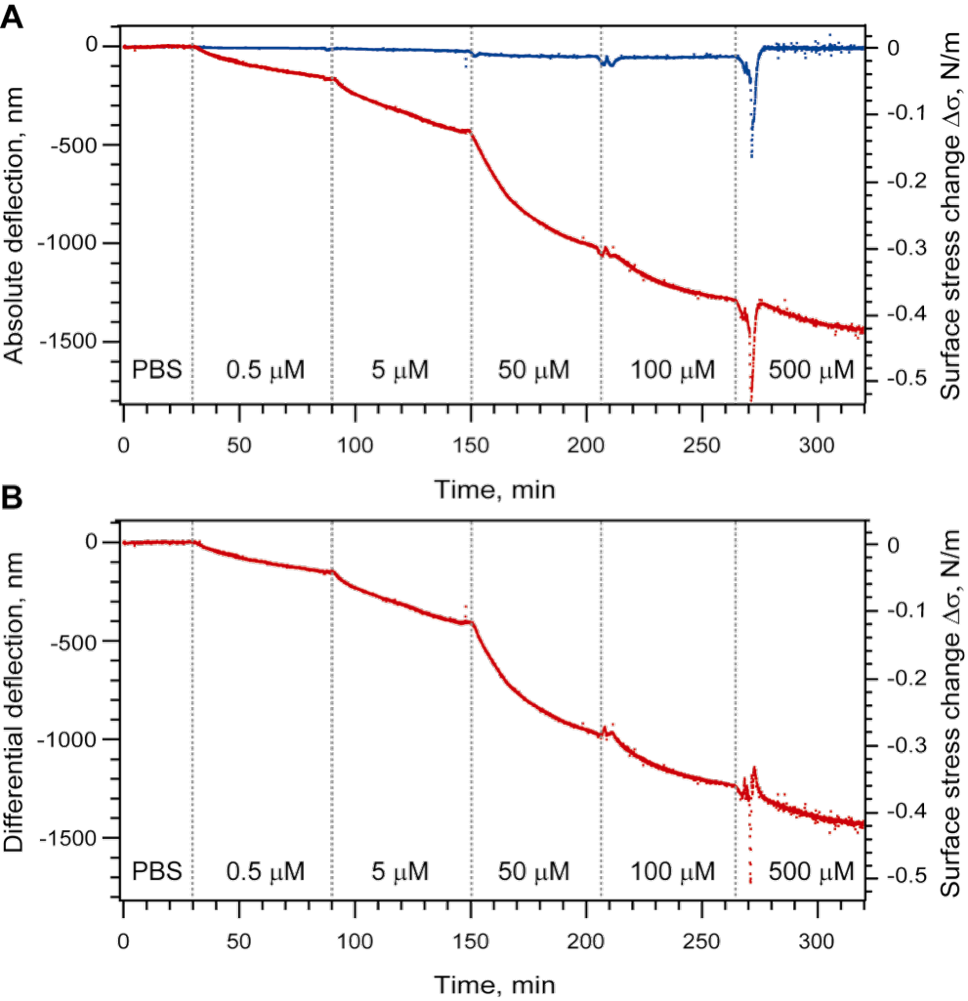
Grafting of mPEG–SH chains on a Au-coated microcantilever surface. (A) Deflection of the sensing (red) and the reference microcantilevers (blue) upon injection of different mPEG–SH dilutions. The vertical dotted lines indicate the beginning of the injection of a new sample concentration. Each curve represents the aligned and averaged data from at least three microcantilevers. (B) Differential deflection of sensing microcantilevers after subtraction of the reference.

After equilibrating the system at 20 °C, freshly prepared PBS solutions containing the mPEG–SH at concentrations of 0.5, 5, 50, 100 and 500 μM were injected in ascending order at intervals of 60 min. As shown in Figure 2A, the chemisorption of mPEG–SH chains generates a compressive force that bends the Au-coated microcantilevers downwards. This behavior is significantly different to that of the cantilever pre-functionalized with EG_4_–C_11_–SH where no adsorption-related bending is observed and confirms that the presence of EG_4_–C_11_–SH blocks any binding of mPEG–SH. At higher concentrations of mPEG–SH (100 and 500 μM), we observed an injection peak related to a change in refractive index. To account for artifacts caused by such changes in refractive index, a differential deflection signal has to be obtained by subtracting the reference signal from the signal of the positive controls (Figure 2B). Based on the 60 min incubation time per mPEG–SH concentration, we find that the differential signal Δ*d* is compressive at all mPEG–SH concentrations and ranges from 130 nm → 400 nm → 980 nm → 1230 nm → 1430 nm for dilutions containing 0.5, 5, 50, 100 and 500 μM, respectively. The differential deflection Δ*d* can be converted into the surface stress change Δσ using Stoney’s equation (see Material and Methods) and corresponds to a Δσ ranging from 30 to 420 mN/m, respectively.

**Figure 3:**
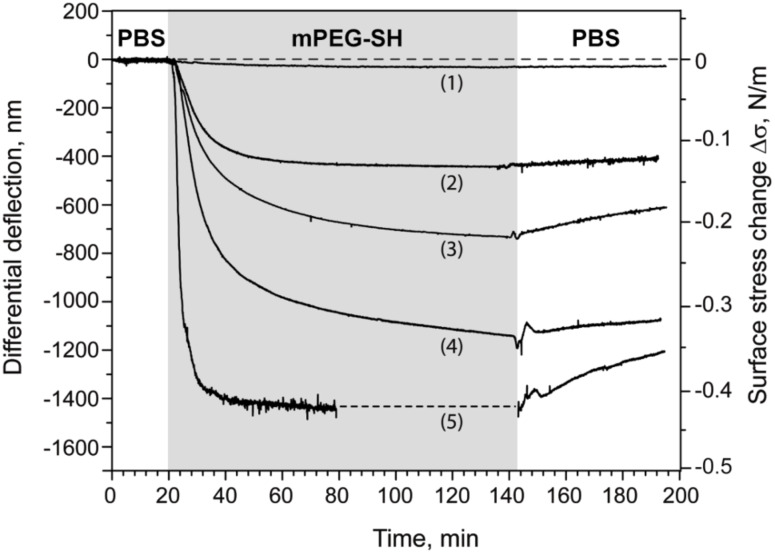
Concentration-dependent grafting of mPEG–SH on Au: overlay view of five binding curves. The grey area indicates the period of injection of mPEG–SH solution. The binding curves were obtained at mPEG–SH concentrations of 0.5 μM (1), 10 μM (2), 50 μM (3), 100 μM (4) and 500 μM (5). Each curve was obtained by subtracting the response of the reference cantilevers from at least two sensing cantilevers from within the same array. The resulting five curves were further normalized with respect to the mechanical properties of the cantilevers used (see Material and Methods section). The dashed line in the curve (5) is the extrapolated saturation signal.

To assess these results, we repeated the measurements in five respective mPEG–SH dilutions (i.e., 0.5, 10, 50, 100 and 500 μM) each using an independent microcantilever array. For consistency, each array had two out of eight Au-coated microcantilevers blocked with EG_4_–C_11_–SH, which served as *in situ* negative controls. The kinetics of PEGylation was monitored for 60–120 min after injection, after which the cell was flushed again with buffer. The resulting differential signal for each concentration-dependent binding curve is displayed in Figure 3 after normalization using a standard mechanical calibration protocol (see Experimental section).

**Figure 4:**
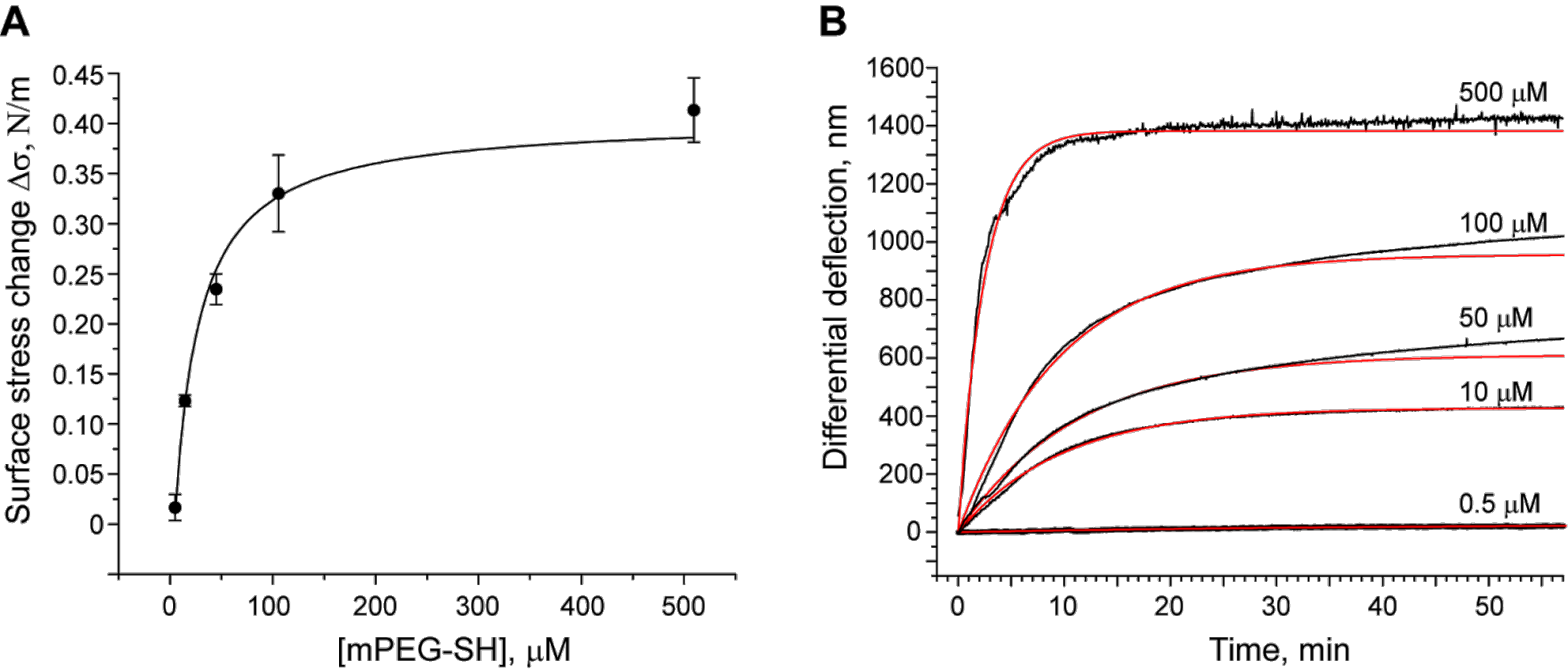
(A) Adsorption isotherm of mPEG–SH on Au. Each point (circle) corresponds to the maximum differential signal observed at the following mPEG–SH concentrations: 0.5, 10, 50, 100 and 500 μM. The solid line represents the Langmuir isotherm fitting curve (R^2^ = 0.982). (B) Representative plots of microcantilever deflection versus time. Kinetic curves (black) were fitted using the exponential Langmuir rate law (red). The curves were fitted for the first 30 min of the binding event and then extrapolated for a longer period of time.

The maximum Δ*d* obtained two hours after the beginning of mPEG–SH injection was converted into the surface stress change Δσ and plotted as a function of concentration (Figure 4A). Here, the maximum nanomechanical surface stress Δσ was found to increase with concentration reaching a plateau at ~410 mN/m. This serves as a clear indication that Δσ is generated by the grafting of mPEG–SH molecules in a concentration-dependent manner. At the highest bulk concentration (Figure 3) we observed a substantial decrease of the binding signal after rinsing the measurement cell with buffer. This could be an indication of physical desorption of unbound mPEG–SH molecules from the top of the PEGylated layer and/or an intercalation of surface-tethered mPEG–SH chains that generates additional surface stress.

The adsorption of surface-active molecules at the interface of a two-phase system (i.e., bulk vs interface) can be described using the classical Langmuir isotherm

[1]
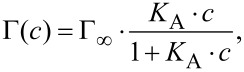


where Γ represents the equilibrium adsorption (surface density) at the bulk component concentration *c*, Γ_∞_ is the capacity of the interface expressing the maximum amount of the component that can be adsorbed and *K*_A_ is the association equilibrium constant. If we consider Γ(*c*) as the equivalent of Δσ(*c*), fitting the data points with Equation 1 results in *K*_A_ = (4.23 ± 0.54) × 10^4^ M^−1^ and a maximum generated stress Δσ of 400 ± 27 mN/m (Figure 4A).

To analyze the PEGylation kinetics on Au in more detail, we fitted the binding curves (Figure 4B) with the Langmuir rate equation that describes the rate of adsorption (or in the current case the signal change *d*) by

[2]
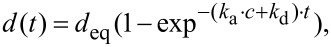


where *c* is the bulk concentration of adsorbing molecules, *d*_eq_ is the equilibrium adsorption (deflection) at this concentration, *k*_a_ and *k*_d_ are the rate constants of association and dissociation processes. Here, we find that the Langmuir fit appropriately describes the experimental curves at PEG concentrations below 50 μM (Figure 4B). Interestingly, we observed a clear deviation from the Langmuir behavior at 50 μM and above. This could arise due to the sensitivity of the microcantilever technique to conformational changes in the PEG molecules that might result from steric effects at higher PEG surface grafting densities, i.e., leading to changes in surface stress. Indeed, conformational effects are not *a priori* accounted for by the Langmuir equation.

**AFM validation of PEG layer formation.** To investigate the local morphology of the PEG layer, we performed AFM imaging and force measurements on the rigid body of a microcantilever array (i.e., its Au surface is similar to that of the microcantilevers) under similar solvent conditions as in the microcantilever experiments. Accordingly, the microcantilever arrays were functionalized by immersion in a PBS solution containing mPEG–SH (500 μM) for two hours and then rinsed with PBS prior to experimentation.

**Figure 5:**
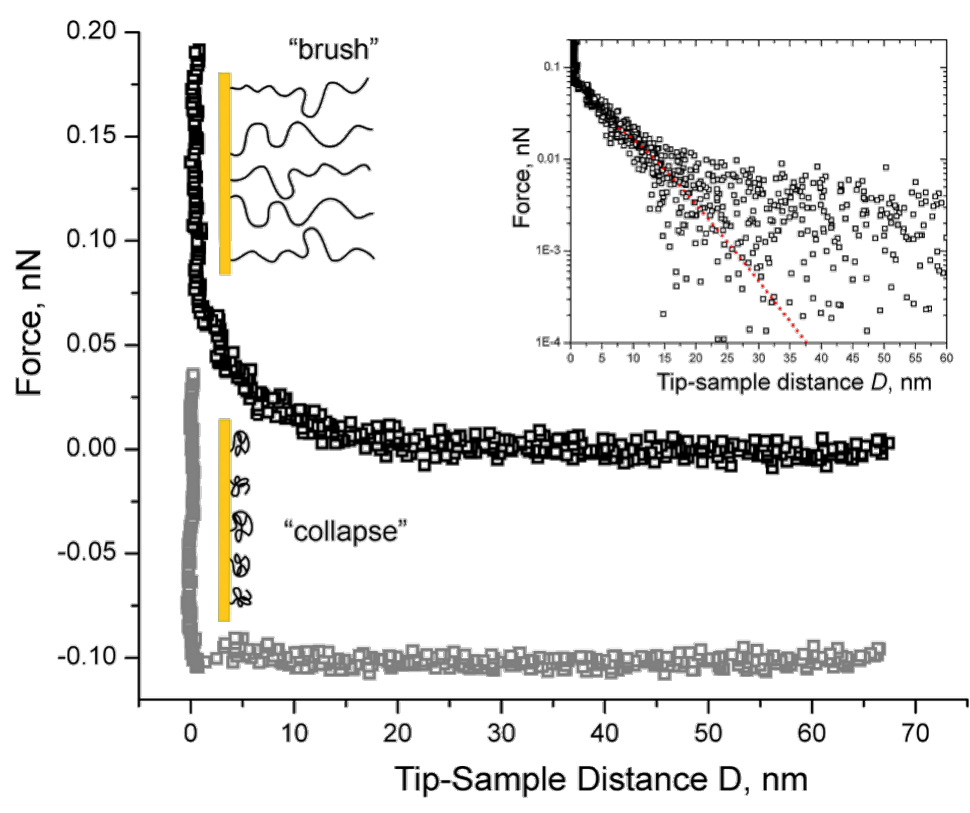
Representative force curves obtained by approaching the AFM tip to the Au surface grafted with 20 kDa mPEG–SH. The exponentially decaying long-range repulsive force in the upper curve indicates that the PEG chains are “brush-like” (black squares) in PBS. Inset: The corresponding fit using eq 3 (red line) gives fitted values of 27.4 ± 0.2 nm and 18.1 ± 0.1 nm for *L* and *s*, respectively. The data collected beyond a certain *D* is scattered being less than the minimum detectable force, which is given by the thermal noise of the AFM-cantilever: *F*_min_ *=* (*k*_B_*T* × *k*_AFM_)^1/2^ ≈ 5 pN. The weak repulsion at ~5 nm in the lower curve indicates that the PEG chains are no longer brush-like in 20% 2-propanol and have collapsed to form a layer that is ~5 nm thick (gray squares). The 0.1 nN offset distinguishes the two force curves as being from separate measurements.

A representative force curve obtained in PBS is shown in Figure 5. Note that *D* = 0, where *D* is the tip-sample approach distance (see Experimental section), is assigned to the region where the force increases infinitely (hardwall repulsion). Here, we measure an exponentially decaying, long-range repulsive force with a detectable onset at a separation distance of *D* ~ 25 nm above the Au surface. This force is characteristic of the compressive response or steric repulsion of a polymer brush as described by the Alexander–de Gennes theory (in a limited range 0.2 < *D*/*L* < 0.9 [10,31–32]),

[3]
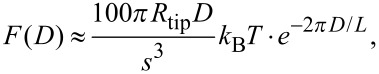


where *F* is the measured force (as a function of *D*), *k*_B_ is Boltzmann’s constant, *T* is the absolute temperature, *R*_tip_ is the radius of the AFM-cantilever tip, *L* is the effective brush height and *s* is the distance between grafting sites. As shown in the inset of Figure 5, Equation 3 provides an appropriate fit to the measured force in PBS where we obtained fitting values of *L* = 27.4 ± 0.2 nm and *s* = 18.1 ± 0.1 nm for *R*_tip_ = 16.6 ± 0.2 nm. When averaged over ~20 force curves obtained with the same tip, we found that the PEG layer has an overall height of *L* = 25.9 ± 2.8 nm with an average inter-chain grafting distance of *s* = 18.0 ± 1.3 nm. This indicates that the PEG chains are in a partially stretched conformation that might result from a mushroom-to-brush transition in the layer (see Discussion).

To further characterize the conformation of the PEG layer, we repeated our AFM measurements in 20% 2-propanol which is a poor solvent for PEG. This is because polymer brushes are known to form more compact “collapsed” clusters, known as pinned micelles, in poor solvents [33]. As we show in Figure 5, the force curves obtained in 20% 2-Propanol do not exhibit any long-range repulsion and are markedly different as compared to the ones obtained in PBS. Instead, a weak repulsion is observed at *D* ≈ 5 nm before the tip comes into hardwall repulsion with the underlying Au surface. Given the lack of repulsion on the AFM tip, this suggests that the PEG chains are no longer in a stretched state and have collapsed to form a layer that is ~5 nm thick.

**Figure 6:**
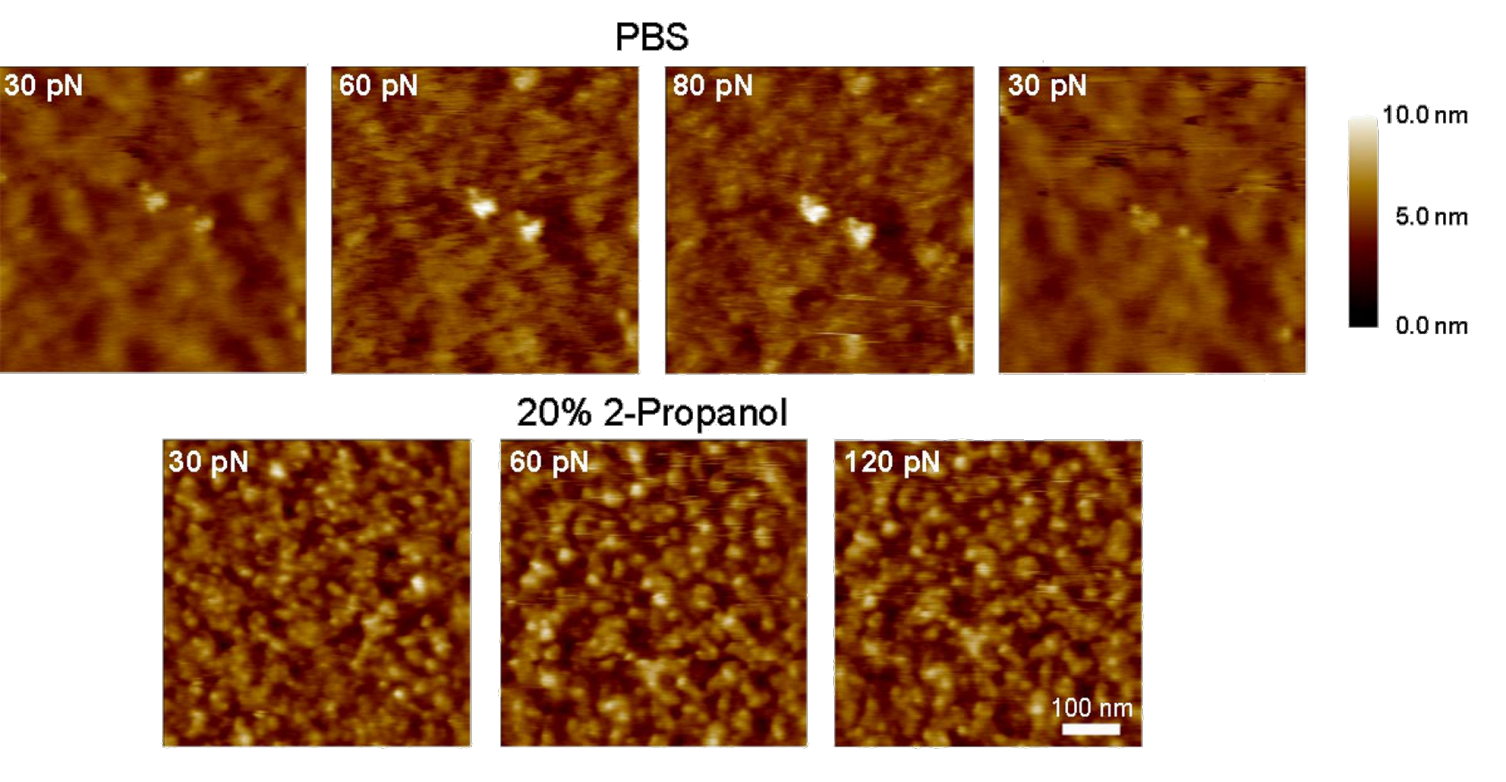
AFM images of Au-tethered PEG-layers obtained at different forces in good solvent (PBS; upper row) and in poor solvent (20% 2-propanol in PBS; lower row) conditions. Images in PBS show an improved resolution of the underlying Au surface (on the same area) as the force set point is increased from 30 → 60 → 80 pN indicating penetration/splaying of the PEG layer by the tip. The Au surface is covered again after the force set point is reduced back to 30 pN. The lower series of images were acquired in 20% 2-propanol and do not show any dependence on the force applied. This effect was similar over different areas on the sample (the 30 pN area is different from the 60 and 120 pN area), and implies that the tethered chains have collapsed under poor solvent conditions. All images were obtained on the same microcantilever array chip. The scale bar is 100 nm.

Figure 6 shows the morphology of the PEG layer obtained in PBS and in 20% 2-propanol at various AFM contact imaging forces. In PBS, we observe a blurry, indistinct image at 30 pN indicating that the AFM tip has not effectively displaced the PEG chains at such low imaging forces. However, increasing the imaging force to 60 and 80 pN results in a splaying and/or penetration of the PEG layer such that the topography of the Au surface becomes more visible at these higher forces. Importantly, the PEG layer appears to conceal the Au surface once the imaging force is reduced to 30 pN. Such behavior contrasts significantly to the situation when PBS is replaced with 20% 2-propanol where the imaging quality is no longer dependent on the applied imaging force. In this case, the AFM image resolution does not change when the imaging force is varied between 30 pN and 120 pN. Here, it is noteworthy that only the underlying Au surface and aggregates of collapsed PEG chains (pinned micelles) are resolved.

**Figure 7:**
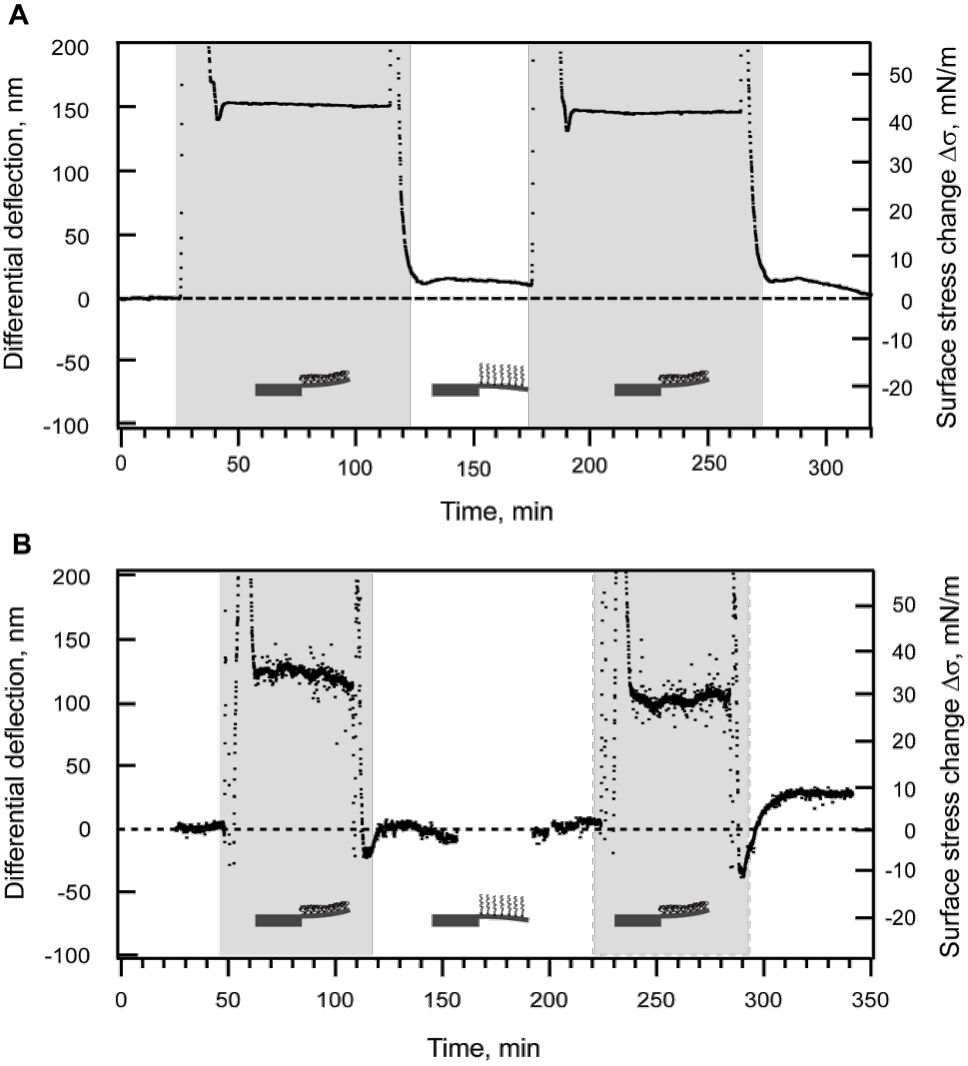
Collapse and restretching of the tethered mPEG–SH-layer observed when switching from good (PBS) to poor solvent conditions, particularly in 20% (A) and 10% (B) 2-propanol in PBS. The duration of each poor solvent injection is shaded in gray. (A) As an *in situ* reference two microcantilevers in the array were blocked with EG_4_–C_11_–SH. (B) As an external reference, a non-functionalized Au-coated microcantilever array was used. The averaged deflection of eight reference cantilevers was subsequently subtracted from the average deflection of eight sensing cantilevers to obtain the differential response.

**Sensing the reversible collapse of a PEG layer.** To test whether our microcantilever sensor is able to resolve the collapse of the PEG layer, we performed measurements under the different solvent conditions similar to those in AFM experiments. A microcantilever array was immersed into 500 μM solution of mPEG–SH in PBS for two hours to ensure that a saturated PEG layer had formed on the microcantilever surface. As an internal reference, two microcantilevers were blocked with EG_4_–C_11_–SH. Interestingly, we observed a marked change in surface stress upon switching between PBS (good solvent) and 20% 2-propanol (poor solvent). An injection of 20% 2-propanol resulted in a tensile (upwards) microcantilever deflection that reached 155 ± 3.5 nm, i.e., equivalent to a surface stress of ~45 mN/m (Figure 7A). This change in differential deflection and, correspondingly, in surface stress was reproducible over consecutive injections of PBS and 20% 2-propanol.

To remove any possible artifacts, we next measured the microcantilever response of two Au-coated microcantilever arrays, one as a reference (non-PEGylated) and the other as a sensing array (PEGylated), respectively. We chose arrays with similar mechanical properties to enable direct quantitative comparisons between their respective results. As before, the arrays were sequentially exposed to PBS and 10% 2-propanol after mPEG–SH functionalization. Here, an injection of 10% 2-propanol caused tensile cantilever bending in both the sensing and the reference arrays (data not shown). However, upon closer examination, the relatively high absolute signal (~100–200 nm) obtained for the reference microcantilevers in 10% 2-propanol was similar to that of the signal acquired from the rigid side bar (see Figure 1) of the microcantilever array (data not shown). We therefore attributed this to differences in the refractive index of the two solvents. Figure 7B shows the resulting curve obtained after subtracting of the averaged reference signal. The collapse of the PEG chains resulted in an upward (tensile) microcantilever deflection of 110 ± 12 nm that corresponds to a generated surface stress change of ~ 32 ± 3.5 mN/m. This is less than the surface stress change related to the 20% 2-propanol mixture in PBS. Nevertheless, the PEG layer collapse was again reversible [32] over several cycles where the return of the signal to its base line level in PBS indicates a return of the collapsed PEG chains into their swelling state.

## Discussion

We have studied the behavior of 20 kDa mPEG–SH “grafted to” Au surfaces using a microcantilever array-based sensor. Consistent with XPS [8], ellipsometry [8,34], QCM [35] and AFM [36] polymer “grafting to” studies, we find that the adsorption profile of mPEG–SH on Au suggests two-regime kinetics [35] of layer formation as characterized by an initial phase of fast chain grafting (first regime) followed by a slow approach that plateaus towards saturation (second regime). We did not observe a third kinetic regime characterizing the so-called mushroom-to-brush transition [37–38]. This kind of transition was observed for systems where segmental adsorption of polymer (i.e., non-covalent polymer-surface interaction) [35] did not occur.

Our analysis of the adsorption isotherm reveals that the generation of surface stress depends on the amount of mPEG–SH bound on Au. Interestingly, we did observe deviations to this trend at high mPEG–SH concentrations. As seen in Figure 4A, the Langmuir adsorption function indicates a lower maximum adsorption than observed in our microcantilever experiments at 500 μM PEG. Apparently, an increase in the bulk concentration appears to promote the physical adsorption of non-tethererd PEG chains on the grafted monolayer after saturation is reached at 100 μM PEG concentration. Indeed, the dissociation profile of the binding curve obtained at 500 μM (Figure 3) seems to support such a view.

The exponential fit of the kinetic curves by the Langmuir rate law (Figure 4B) demonstrates a good correlation between experiment and theory at concentrations less than 50 μM. Apparently, at these low concentrations/surface densities we predict a formation of non-overlapping “mushrooms” (see below) that do not interact with each other on the surface. With increasing grafting density, the distance between grafting sites decreases so that tethered chains come into contact and start to overlap. The corresponding conformational changes and rearrangement of the molecules on the surface are reflected in the microcantilever deflection signal. Bearing in mind that the microcantilever array technique measures changes in surface stress, our results at higher PEG concentrations (> 50 μM) imply that conformational changes have occurred between the grafted PEG chains after the initial binding to the Au surface due to the increase of PEG surface density.

**Figure 8:**
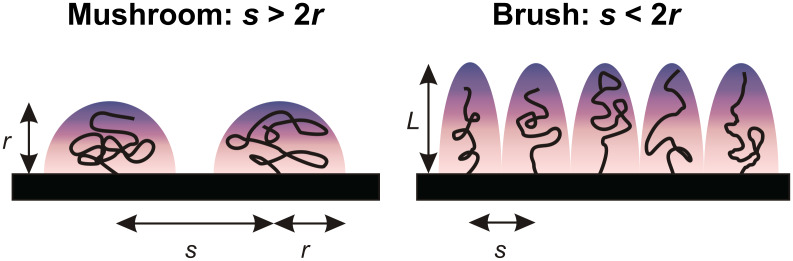
Mushrooms versus Polymer Brushes: a sketch illustrating how surface-tethered polymer chains can take on either “mushroom” -like or “brush”-like molecular conformations, depending on how closely packed the polymer chains are. The mushroom regime occurs when the distance between neighboring chains *s*, is greater than twice the radius of the polymer. The brush regime is encountered when *s* < 2*r* and the polymer chains are extended away from the surface at a height of *L*. The shaded areas emphasize the stochastic nature of the polymer chains where each chain has a high probability to occupy all positions within a given volume.

Depending on the distance between grafting sites *s*, surface-tethered polymer chains can take on either “mushroom”-like or “brush”-like molecular conformations [39–41]. At low surface densities, adjacent polymer chains form mushrooms that do not laterally overlap, whereas elongated brushes form at higher grafting densities. The mushroom regime is often invoked when *s* is greater than twice the Flory radius *R*_F_ of the polymer (*s* > 2*R*_F_) while the brush regime is encountered when *s* < 2*R*_F_ [9] (see Figure 8). The Flory radius is considered as an estimated size of a polymer coil. Given that *R*_F_ ≈ *lN*^3/5^ where *l* is the monomer length and *N* is the degree of polymerization [39], the size of a single 20 kDa mPEG–SH chain is given by *R*_F_ ≈ 14.4 nm as calculated from *l* ~ 0.37 nm [42] and *N* ~ 450. Considering the height (*L ~* 26 nm) of the PEG layer and the grafting distances (*s ~* 18 nm) as compared to 2*R,* the Alexander–de Gennes fit to our AFM data in PBS suggests that the PEG molecules have formed either a saturated layer of mushrooms or a brush-like layer in the “weak overlap“ regime (*R*_F_ < *s* < 2*R*_F_) [41,43] on the Au surface. Notably, this finding is in close agreement with the inter-chain grafting distance of *s* ~ 18 nm (calculated from a grafting density of ~0.003 chain/nm^2^) reported for PEG chains grafted onto silicon substrates from a 1 mM solution of PEG (20 kDa) [7]. Our findings are also consistent with the observation that the grafting densities necessary for the formation of a dense polymer brush are less likely obtained by the “grafting to” approach [42].

AFM images of the PEGylated Au surface taken under different solvent conditions reinforce the interpretation that a brush-like layer has formed. In PBS buffer (good solvent), we find that the surface topography is strongly dependent on the AFM scanning force used. At ~30 pN, the underlying Au surface is obscured by the presence of the PEG brush due to steric repulsive forces that act on the AFM tip [32]. Only when the force is increased (to 60 or 80 pN) does the AFM tip penetrate the PEG brush to resolve the underlying surface structure (Figure 6). In contrast, the Au surface topography does not change regardless of the scanning force used in 20% 2-propanol (poor solvent). This is due to the collapsed conformation of tethered PEG chains that offers minimal resistance against the AFM tip. The influence of solvent quality on the AFM images can be understood by comparing the tip-sample interaction forces obtained in PBS and 20% 2-propanol, respectively (Figure 5). Specifically, the thickness of the PEG layer (defined by the onset of repulsion) reduces from 26 nm in the brush-like state to 5 nm in the collapsed state.

It is noteworthy that the microcantilever arrays are also sensitive to the reversible collapse [32] of tethered PEG chains by cycling through injections of PBS and 20% 2-propanol, respectively. The nanomechanical hallmark of the PEG chains switching from a brush-like to a collapsed conformation (de-swelling) is described by a reproducible tensile surface stress change with a deflection of ~110–150 nm which equates into a generated stress of ~35–45 mN/m. These results were reproducible with respect to both *in situ* and external references, as well as surfaces consisting of either bare Au or a Au surface blocked with a self-assembled EG_4_–C_11_–SH monolayer. The resulting bending is similar to Bumbu et al. [29], who showed that the de-swelling of dense poly(methyl methacrylate) brushes “grafted from” a silicon microcantilever generated tensile surface stress.

Our results demonstrate that microcantilever array sensor technology can be used as an *in situ* technique with the capability to characterize both qualitative and quantitative processes that occur during and after polymer layer formation. In comparison to other surface characterization tools, conformational changes and kinetic measurements can be implemented within a single nanomechanical platform. Another benefit is that it can be applied to monitor solvent-dependent conformational changes and nanomechanical properties in a polymer layer in real-time without having to probe the surface directly (for instance by AFM). When used in conjunction with protein adsorption studies, this may have specific applications as integrated (and miniaturizable) process sensors**.** More generally, this particular capability of microcantilever sensors may provide new insight into the biochemical and nanomechanical properties of biopolymers *in vitro.* For instance, it is estimated that ~30% of the cell is composed of natively unfolded/intrinsically unstructured proteins [44] and that several of these are anticipated to function in a brush-like conformation. These include the natively unfolded Phe-Gly (FG)-domains of the nuclear pore complex [45], microtubule associated proteins [46], neurofilaments [47] etc.

## Conclusion

Microcantilever arrays have been used to monitor PEGylation kinetics and nanomechanical changes in the grafted PEG layer in real time. Surface stress measurements indicate that the PEG chains adopt a brush-like confirmation at higher bulk concentrations. This was confirmed by AFM images and force measurements of the PEGylated Au surface in different solvent conditions. As opposed to the brush-like conformation in PBS (good solvent), a 20% 2-propanol PBS solution caused the PEG chains to collapse into more compact structures. The collapse was validated and shown to be reversible using microcantilever sensors. Our work suggests how microcantilever sensors may be applied to studying the kinetics and nanomechanics of natively unfolded proteins and other brush-forming molecules.

## Experimental

**Materials.** Phosphate-buffered saline (PBS) buffer (0.067 M phosphate, 0.15 M NaCl, pH 7.4) was prepared by dissolving PBS-tablets purchased from Sigma-Aldrich (Buchs SG, Switzerland) in HPLC-grade water (Fluka, Buchs SG, Switzerland) according to the supplied protocol. 2-Propanol was obtained from Merck AG (Altdorf, Switzerland). Methoxy(polyethylene glycol) thiol 20000 (mPEG–SH, MW ~ 20000) was purchased from Laysan Bio Inc (Arab, AL). (1-Mercapto-11-undecyl)tetra(ethylene glycol) (EG_4_–C_11_–SH, MW ~ 380.5) was obtained from Asemblon Inc (Seattle, WA), diluted in ethanol to an end concentration of 10 mM and stored at +4 °C.

**Coating of microcantilever arrays with Au.** Arrays of eight silicon microcantilevers (500 μm long, 100 μm wide and 1 μm thick with *k*_cantilever_ ~ 0.03 N/m) were fabricated at the IBM Zurich Research Laboratory (Figure 1A). Before Au coating, the arrays were cleaned in Piranha solution (30% H_2_O_2_/96% H_2_SO_4_ = 2:1 v/v) for 15 min, rinsed three times with water followed by ethanol and dried in air. The upper sides of microcantilevers were coated with a 2 nm-layer of Ti followed by a 25 nm thick Au layer without breaking the vacuum. Deposition of metal layers was performed in an EVA 300 electron beam evaporator (Alliance Concept, Cran Gevrier, France) at an evaporation rate of 0.1 nm/s. Au-coated arrays were used immediately or stored under argon atmosphere for a maximum of two days.

**Microcantilever functionalization.** To study the grafting of mPEG–SH on Au, two microcantilevers were blocked with EG_4_–C_11_–SH and used as an internal reference. To this end, 10 mM stock solution of EG_4_–C_11_–SH was diluted in water to a final concentration of 1 mM. Seven drops (0.1 nL/drop) of the dilution were dispensed along microcantilever surfaces using a modified ink-jet spotting system [48] MDP705L (Microdrop Technologies, Norderstedt, Germany) utilizing a nozzle with an inner diameter of 70 μm. After evaporation of the dispensed solution, the array was thoroughly rinsed with PBS buffer and mounted in the measurement chamber pre-equilibrated with PBS. Thus, the resulting array consisted of two microcantilevers with blocked Au surfaces and six microcantilevers with functional Au layers for mPEG–SH grafting.

For measurements of the PEG-brush collapse using an internal reference, two microcantilevers in the array were blocked with EG_4_–C_11_–SH self-assembled monolayers (SAMs) using an ink-jet spotter as described above. To coat the remaining six microcantilevers with mPEG–SH, the entire array was immersed in 0.5 mM polymer solution in PBS for 2 h at room temperature and finally washed with PBS.

For experiments on the collapse using an external reference, two separate Au-coated microcantilever arrays were applied: the sensing array was coated with mPEG–SH by immersing the entire array in 0.5 mM mPEG–SH solution (PBS) for 2 h at room temperature, rinsed with PBS and then mounted in the measurement chamber; the reference array was not functionalized.

**Sensor instrument.** All measurements were performed on a home-built static mode device for detecting the microcantilever deflection with an integrated optical read-out (Figure 1B). The bending of microcantilevers was detected by reflection of an external laser beam (λ = 760 nm) focused at the microcantilever apex. The instrument enables monitoring the deflection of all eight microcantilevers in parallel in a time-multiplexed manner. All measurements were performed in a temperature-controlled box under a steady buffer flow. Data acquisition hardware, temperature regulation and a syringe pump for buffer and sample injection were controlled using LabView software.

**Mechanical calibration of microcantilevers.** Prior to the experiment, the chamber was flushed with PBS and the microcantilevers were calibrated for their mechanical homogeneity using a heat pulse of 2.5 °C for 1 min (mechanical test). Only cantilevers with a maximum deflection difference within 10% were considered for further experiments. The mechanical test was used to determine normalization factors to correct for mechanical inhomogeneity between different microcantilevers.

**Static mode measurements and data processing.** PBS buffer solutions utilized in static mode measurements were filtered (0.2 μm filter, Millipore) and degassed in vacuum for at least 10 min. The PBS/2-propanol mixture was degassed by sonication in a water bath for 30 min. All measurements were performed at +20 °C under constant flow at a rate of 5 or 10 μL/min.

The collected data were processed using NOSEtools [49] software based on the Igor Pro 6 platform. The microcantilever data sets were aligned and normalized using the normalization factors (see Mechanical calibration of microcantilevers) as described in detail previously [50]. An average deflection was calculated for both, the reference and the sensing microcantilevers. The differential signal was calculated by subtraction of the reference data. Data obtained in two separate measurements were compared with each other with regard to the mechanical tests and corresponding calibration coefficients. The induced surface stress Δσ change (N/m) was calculated from the microcantilever bending Δ*d* using Stoney’s equation [51]:

[4]
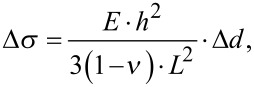


where *E* is the Young modulus of silicon (170 GPa), *h* is the thickness of the microcantilever (10^−6^ m), ν is the Poisson ratio (0.23) and *L* is the microcantilever length (500 × 10^−6^ m).

**AFM force measurements and imaging.** AFM contact mode imaging and force measurements were performed in either PBS buffer or in a binary mixture of 20% 2-propanol in PBS (termed 20% 2-propanol) at room temperature using a Multimode-Nanoscope IIIA controller (Veeco, Santa Barbara, CA) equipped with a 120 μm J-scanner and a standard liquid cell. The Au-coated microcantilever arrays (i.e., substrates), were functionalized by immersion in a mPEG–SH dilution (500 μM) in PBS for two hours at +20 °C. Prior to each measurement the system was allowed to equilibrate for 1 h after which the drift observed within individual force measurements (single approach-retract cycles) was negligible. Rectangular-shaped Si_3_N_4_ AFM-cantilevers (Biolever, Olympus/OBL, Veeco) with V-shaped tips were used in all measurements. Spring constant calibrations typically fell within a 20% margin of error from the nominal spring constant of 0.005 N/m. The radius of curvature of each AFM-cantilever tip (*R*_tip_) was evaluated using scanning electron microscopy (SEM) after the AFM experiments. The AFM data shown was obtained using a tip with *R*_tip_ = 16.6 ± 0.2 nm. The force acting on the tip is given by the linear relation between the AFM-cantilever deflection and *Z* (when the tip is in hardwall repulsion) multiplied by the AFM-cantilever spring constant *k*_AFM_. For the force curves the *F* vs *Z* data were converted to *F* vs tip - sample approach distance (*D*) by further subtraction of the AFM-cantilever deflection from *Z* [32]. The AFM contact mode images were obtained at a scan rate of ~1.5 µm/s.
